# The Biogeography of Apicomplexan Parasites in Tropical Soils

**DOI:** 10.1002/ece3.71478

**Published:** 2025-06-02

**Authors:** Rachel M. Shepherd, Angela M. Oliverio

**Affiliations:** ^1^ Department of Biology Syracuse University Syracuse New York USA

**Keywords:** Apicomplexa, microbial eukaryotes, parasites, protists, soil biogeography, soil microbial ecology

## Abstract

Parasitic protists such as Apicomplexa, an abundant group of soil protists, contribute to ecosystem processes and nutrient cycling in belowground soil systems through their obligate symbioses with soil Metazoa. Yet despite the importance of soil parasites, the biodiversity and biogeography of Apicomplexa in belowground systems remain poorly characterized. Leveraging 205 soils collected across a rainfall gradient spanning the isthmus of Panama, we sought to understand the distribution of soil Apicomplexa lineages and how abiotic (e.g., soil and climatic) and biotic (e.g., soil Metazoa) factors relate to their diversity and structure. Apicomplexa were highly heterogeneous across the samples and comprised 30% of the soil protist community on average. Soil pH, along with phosphorus and magnesium, best explained the overall distribution of Apicomplexa. Soil Metazoa distributions also corresponded to Apicomplexa distributions, and many Metazoan taxa co‐occurred with particular Apicomplexa, which may reflect ecological interactions (such as parasitism) or shared habitat preferences. These results highlight the potential roles of both soil and climatic variables and putative hosts in structuring parasite distributions in belowground tropical systems. Our work builds a broader understanding of Apicomplexa biodiversity in tropical soils and sheds light on environmental factors that may contribute to shaping their distribution in belowground systems. These results help inform our understanding of the importance of parasites in tropical forest soils.

## Graphical abstract

We investigate the belowground biogeography of an important yet poorly described group of soil parasites, the Apicomplexa. We found both biotic and abiotic drivers that contribute to shaping the diversity and distribution of dominant Apicomplexa in tropical soil systems.

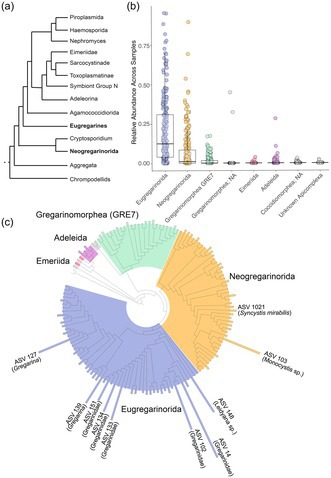

## 1 Introduction

Protists (i.e., microbial eukaryotes) are increasingly recognized for their ecological contributions to belowground systems (Geisen et al. 2017, 2018). Globally, soil protists occupy a diversity of functional roles from primary producers to consumers to parasites (Oliverio, Geisen, et al. 2020; Adl and Gupta 2006; Geisen et al. 2018). Collectively, these contributions regulate belowground food web dynamics, nutrient cycling, and corresponding shifts in aboveground ecosystem dynamics. For example, photosynthetic protists likely provide an important carbon input in soils (Schmidt et al. 2016; Seppey et al. 2017). Bacterivorous and mycophagous protists release plant‐available nutrients via microbial predation (Bonkowski and Clarholm 2012; Clarholm 1985; Hunt et al. 1987). There is also increasing evidence from environmental surveys (Mahé et al. 2017; Oliverio, Geisen, et al. 2020; del Campo et al. 2019) that parasitic protists can also be extremely abundant in soils globally. Despite their ubiquity, the diversity and ecology of parasitic protists remain poorly understood (Geisen and Bonkowski 2018; Grossmann et al. 2016).

In belowground systems, soil Metazoa host a suite of parasites, including Apicomplexa, a clade of spore‐forming protists. Recent surveys of soil protists using environmental barcoding approaches have demonstrated that Apicomplexa are an abundant group of soil‐associated parasites globally (Oliverio, Geisen, et al. 2020; Mahé et al. 2017; Geisen et al. 2018; Li et al. 2023). They are particularly diverse and abundant in tropical soils (Wu et al. 2022), where they can comprise > 90% of the protist reads in a soil (Mahé et al. 2017). In aboveground ecosystems, parasite regulation of host population sizes has profound effects on trophic interactions by affecting competition and biodiversity (Hatcher et al. 2006; Hudson and Greenman 1998; Lafferty et al. 2008). It is likely that soil parasites also contribute to the regulation of key belowground processes.

Gregarines (i.e., Apicomplexa: Gregarinomorphea) parasitize soil arthropods, a dominant group of soil fauna that transform plant litter and are considered ecosystem engineers (Culliney 2013). Gregarines have also been linked to other soil metazoan groups including annelids (Sapkota et al. 2020; Wang et al. 2020) and nematodes (Li et al. 2023; Desportes and Schrével 2013). Intriguingly, gregarines are often obligate endoparasites but can span the symbiotic spectrum based on their effect within their host (Rueckert et al. 2019; Desportes and Schrével 2013). Although a handful of Apicomplexa are extremely well studied as parasites of humans or other vertebrate hosts (Rueckert et al. 2019; Morrison 2009), most lineages that parasitize soil Metazoa remain unknown or poorly described, and the abiotic and biotic factors that control the environmental distributions of Apicomplexa lineages remain unclear. As soil Metazoa contribute to the regulation of key functions including nutrient cycling (Scheu 2002) and plant health (Elmquist et al. 2023; Menta and Remelli 2020), such analyses are critical to advancing our understanding of the role of parasites in belowground ecosystems.

Although there is increasing evidence that Apicomplexa comprise a substantial portion of the soil microbiome in tropical ecosystems, their spatial distributions in relation to belowground edaphic properties and biotic factors remain poorly characterized. Previous research has shown that, on a global scale, Apicomplexa distributions are linked to precipitation and temperature (Oliverio, Geisen, et al. 2020). Other studies have shown that Apicomplexa abundance can vary by land use, pH, total nitrogen, total carbon, and mean annual precipitation (MAP) (Li et al. 2023; Santos et al. 2020; Aslani et al. 2024) and correspond to Metazoa diversity (Singer et al. 2020). However, it remains unclear what factors drive soil parasite abundance and how predictable their distributions are in tropical forests, the ecosystems in which they are most abundant globally. There is also an extreme diversity of invertebrates in tropical forest soils (Basset et al. 2012; Porazinska et al. 2012). Yet, to what extent the diversity and distribution of parasites correspond to the diversity and distribution of soil Metazoa has not been investigated.

Here, we evaluated a series of questions in an effort to advance our understanding of the biogeography of Apicomplexa, a dominant group of belowground soil parasites: (1) What are the dominant lineages of Apicomplexa found in tropical soils? (2) How ubiquitous are individual lineages? (3) How predictable is the composition of soil Apicomplexa across spatial scales and environmental gradients in tropical forest soils? (4) To what extent does the distribution of Apicomplexa correspond to the distribution of metazoan lineages? To address these questions, we analyzed Apicomplexa lineages from a collection of 205 soils collected across the isthmus of Panama. We coupled highly resolved spatial and biogeochemical data with 18S rRNA amplicon gene sequencing to assess the abundance and distribution of both Apicomplexa, a dominant clade of soil parasites, and soil Metazoa, focusing on the invertebrate lineages that are known to host Apicomplexa parasites.

## 2 Materials and Methods

### 2.1 Study Site and Sample Collection

**FIGURE 1:**
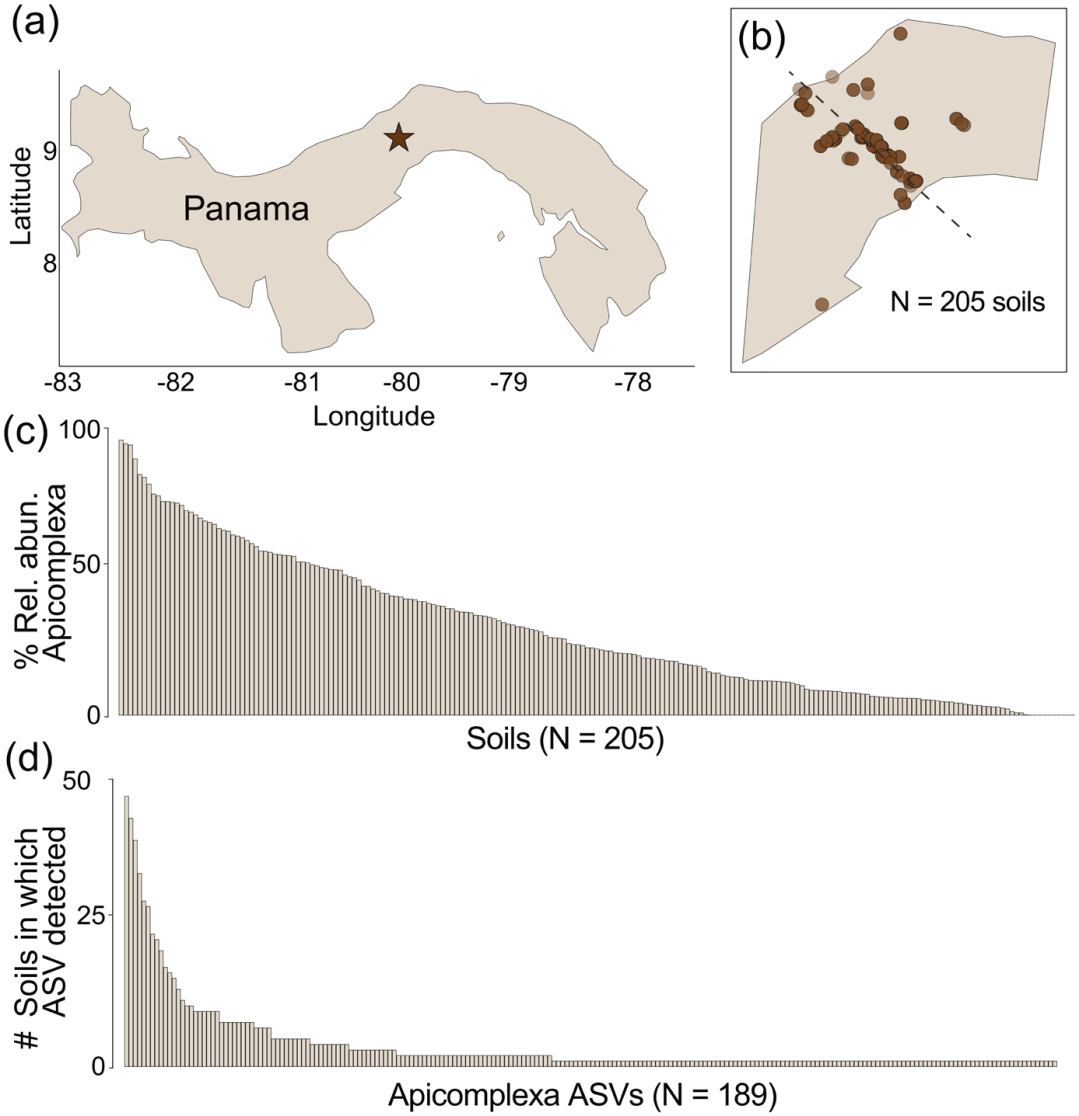
Description of the distribution of soil samples and corresponding Apicomplexa populations across all samples analyzed in this study. (a) Map of Panama, star denotes study area. (b) Sampling locations (*N* = 205 soils for samples containing both protist and metazoan taxa). (c) Bar charts displaying the percent relative abundance that Apicomplexa comprises for each sample out of the entire protist community and (d) the number of soils in which each Apicomplexa ASV was detected.

To characterize Apicomplexa and Metazoa lineages in tropical soils, we used soils that were previously collected and described (see Oliverio, Bissett, et al. 2020 for full details on the soil collection efforts, DNA extraction, and sequencing methods). In brief, mineral soil samples were collected from the top 0–10 cm of forest census plots in Panama (Figure 1a,b) during the wet season between July and December 2017 from 64 previously established plots (Oliverio, Bissett, et al. 2020). The plots span a large gradient of MAP and vary in soil properties including pH, available P (Oliverio, Bissett, et al. 2020), and micronutrients (Shepherd and Oliverio 2024). From each of the sites, five soil cores were collected (Total *N* = 320) from an area of 30 × 30 cm, and each of the soil cores was made up of multiple 1‐in. cores composited to make each sample.

Biogeochemical variables were also characterized for each sample (Table S1). Extractable aluminum (Al), calcium (Ca), iron (Fe), potassium (K), magnesium (Mg), manganese (Mn), sulfur (S), and zinc (Zn) were assessed via Mehlich‐III extraction (Mehlich 1984). Total C was determined by dry combustion, and resin P (a measure of available P) was determined by extraction with anion exchange membranes (Turner and Romero 2009). Latitude and longitude at each sample location were used to obtain information on mean annual temperature and MAP from the WorldClim database (Hijmans et al. 2005). Normalized Difference Vegetation Index (NDVI) served as a proxy for net plant primary productivity (Pettorelli et al. 2005). Measures of resin P, total C, dissolved organic carbon (DOC), K, NH_4_^+^, NO_3_^−^, Ca, Mg, S, Mn, Zn, Fe, and Al were log‐transformed for analyses.

### 2.2 Bioinformatics

To describe the Apicomplexan and Metazoan lineages across the soils surveyed, we sequenced a portion of the 18S rRNA gene, using methods described previously (Oliverio et al. 2018; Oliverio, Geisen, et al. 2020). We processed raw reads into amplicon sequence variants (ASVs) using the DADA2 pipeline (Callahan et al. 2016, version 1.24.0), as per Webster et al. (2021). We removed sequences with unassigned bases (e.g., Ns) and trimmed primers. Forward sequences were quality filtered with the following parameters: truncLen = 250, maxEE = 2, and truncQ = 2. Reverse reads were not included due to poor quality. After quality filtering, sequence variants were inferred with the DADA2 algorithm. We removed chimeras and assigned taxonomy as per the DADA2 pipeline, using the PR2 database (v4.14.0; Guillou et al. 2013). The table was filtered to remove any reads that were assigned to Streptophyta, fungi, or prokaryotes. Reads unassigned at the phylum level were also removed. We also removed samples with incomplete biogeochemical profiling. We rarefied the samples to 1006 reads resulting in a sample set of 205 soils included in downstream analyses. We detected Apicomplexa in 171 of the 205 soils.

### 2.3 Statistical Analyses

To assess the distribution of Apicomplexa among all protists in the samples, we filtered out the metazoan taxa from the table (leaving only the protists; 1970 ASVs). We ran descriptive statistics (i.e., mean, range, and standard deviation) on the relative abundance of Apicomplexa taxa. We also calculated how many times each Apicomplexa ASV (189 ASVs) occurred across the samples. We assessed the distribution of the relative abundance of the Apicomplexan orders across samples. To highlight the phylogenetic diversity of Apicomplexa in the samples, we built a tree using their 18S rRNA gene amplicon sequences. We compiled the sequences and used Silva Incremental Aligner (SINA v1.2.12) “compute tree” (Denovo and FastTree parameters) to align the nearest neighbors (Pruesse et al. 2012). We used trimAl to trim the gaps in the alignment (gap threshold = 0.2; Capella‐Gutiérrez et al. 2009). We built a tree using RAxML‐HPC BlackBox (via CIPRES) with the default parameters for nucleotide trees (Miller et al. 2010; Stamatakis 2014). We visualized the tree with iTOL (Interactive Tree of Life), where we annotated tips based on Apicomplexa class and the mean relative abundance of the ASV across samples (Letunic and Bork 2007). To highlight how the lineages abundant here nest within the broader diversity of Apicomplexa, we also created a cartoon tree based on the work of del Campo et al. (2019).

We next sought to assess how many of our detected Apicomplexan groups may have either a global or more restricted distribution. To do this, we queried the metaPR2 database (Vaulot et al. 2022) for studies that also used the v4 region to characterize soil protists. In total, there were 312 samples from three studies: Mahé et al. (2017) (tropical soils), Seppey et al. (2020) (temperate soils from Switzerland), and Bates et al. (2013) (global soils). We filtered each ASV table to include only Apicomplexa hits and then created a merged set of ASVs from all studies. The phylogenetic tree was built as described above (RAxML‐HPC BlackBox) and the source study was annotated for each ASV.

Our next objective was to assess the abiotic and biotic factors contributing to the distribution of Apicomplexa across tropical forest soils. We calculated the ASV richness and ASV Bray‐Curtis community dissimilarity for the overall Apicomplexa and metazoan taxa (as predictor variables) and on the predominant taxa within each lineage (for Apicomplexa: Eugregarinorida and Neogregarinorida; for Metazoa: Annelida, Arthropoda, and Nematoda). We used Spearman's correlations to determine the relationship between richness and each predictor variable. To assess the correlation between community composition and predictor variables, we calculated the Euclidean dissimilarity matrices of the abiotic predictor variables to compare to the ASV dissimilarity matrices using Mantel tests with Spearman correlations (Oksanen et al. 2022). The correlation of community composition of Apicomplexa and metazoan lineages required subsetting so that each sample included at least one ASV of the specified taxa. Subset sample sizes were as follows: Apicomplexa and Metazoa (*N* = 171); Apicomplexa and Annelida (*N* = 50); Apicomplexa and Arthropoda (*N* = 65); Apicomplexa and Nematoda (*N* = 129); Eugregarinorida and Metazoa (*N* = 168); Eugregarinorida and Annelida (*N* = 48); Eugregarinorida and Arthropoda (*N* = 65); Eugregarinorida and Nematoda (*N* = 127); Neogregarinorida and Metazoa (*N* = 107); Neogregarinorida and Annelida (*N* = 36); Neogregarinorida and Arthropoda (*N* = 40); Neogregarinorida and Nematoda (*N* = 87). To determine the subset of abiotic variables that best explained variation in community composition for each major group, we used the ‘bioenv’ function (*vegan* package; Oksanen et al. 2022) including only variables that had significant (*p* < 0.05) Spearman correlations.

We built co‐occurrence networks with the protist and Metazoa taxa that were observed in at least 5% of the samples. We used Spearman correlations (‘rcorr’ function in *Hmisc* package; Harrell Jr and Dupont 2019) on the ASV relative abundances. Significant correlations (*p* < 0.05; rho > 0.2) between ASVs were retained and visualized with Gephi (v 0.10.1; Bastian et al. 2009). We used modularity 2.0 on the network leaving 2 distinct subnetworks. In the subnetworks, we pulled out the connections that were specific to Apicomplexa and metazoan taxa and visualized the sum of connections (i.e., the number of edges) between each subnetwork. We also correlated the *z*‐scored relative abundances (‘zscore’ function in *OTUtable* package; Linz 2018) with abiotic parameters using Spearman correlations. To provide an example of these relationships, we visualized the relationship between the *z*‐scored relative abundance and pH. We similarly assessed if there were connections between Apicomplexa and other protist taxa.

## 3 Results and Discussion

### 3.1 What Apicomplexa Lineages Are Dominant in Tropical Soil Systems?

Apicomplexa were widespread across tropical soils and often comprised a large portion of the protist community. We detected Apicomplexa in 83% of soils (171 out of 205 soils) at > 1% relative abundance. On average, Apicomplexa comprised 30% of the overall protist composition when present in a given sample. One striking finding from our assessment was the remarkable heterogeneity in Apicomplexa composition across the soils. Their distribution was highly heterogeneous, ranging from 0% to 96.8% relative abundance in individual soils sampled (Figure 1c). In most soils, only one to a few Apicomplexa parasites were detected. The count of Apicomplexa ASVs observed in soil ranged from 1 to 49 ASVs, with a median of 1 (Figure 1d). Most Apicomplexa ASVs detected were also highly soil specific. Surprisingly, over 50% of Apicomplexa ASVs (*N* = 117) only occurred in a single sample. The low overlap observed across soils may be in part due to sequencing depth, and with additional sequencing more shared lineages may resolve. However, it may also suggest that many Apicomplexa lineages are restricted in their distributions or highly host specific, reflecting the extensive diversity of soil Metazoa in tropical soils (Basset et al. 2012; Porazinska et al. 2012).

**FIGURE 2:**
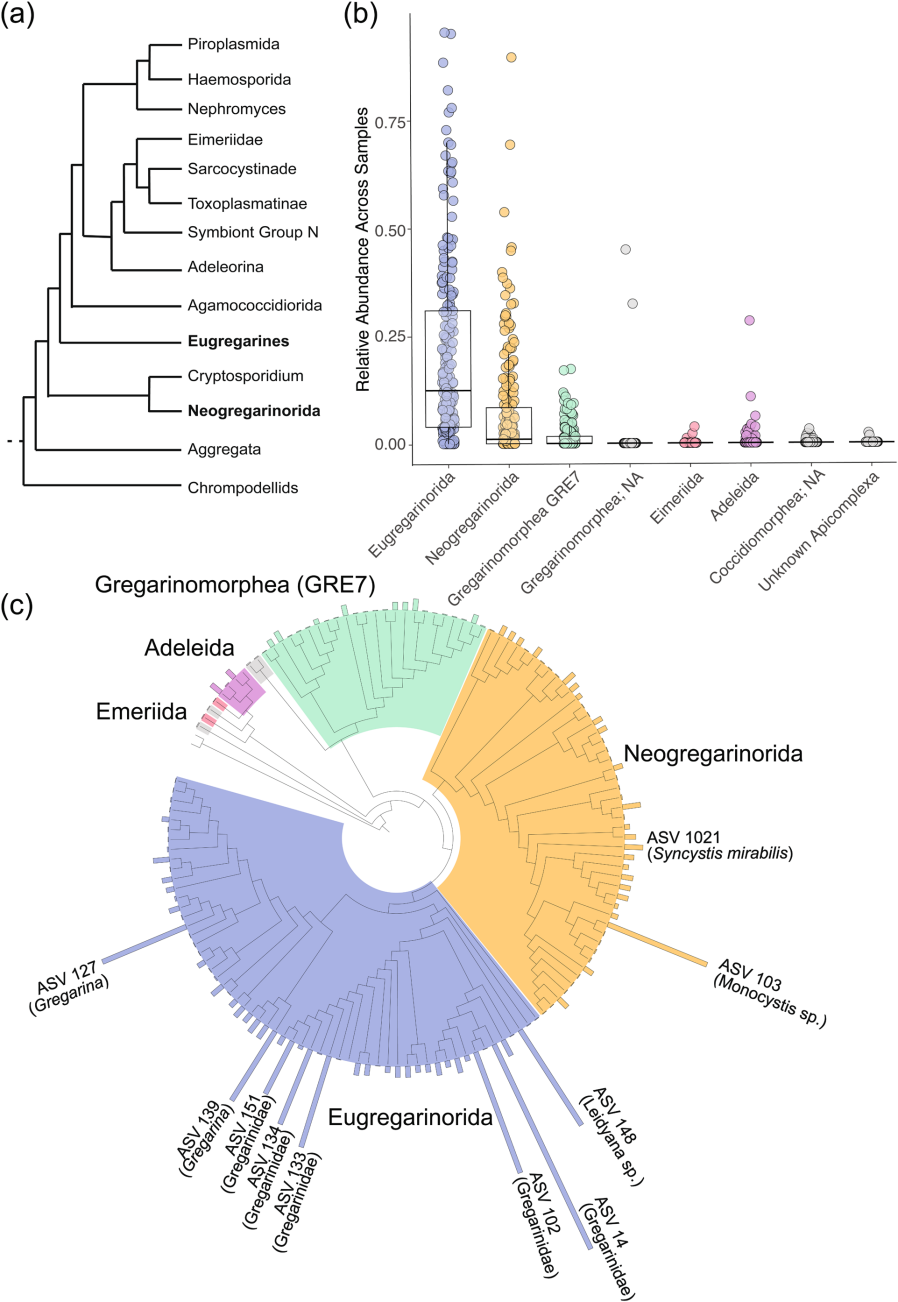
Distribution of Apicomplexa taxa across the sampling sites. (a) Cartoon tree depicting the major lineages of Apicomplexa based on del Campo et al. (2019). (b) Boxplot with jittered raw data points illustrating the percent relative abundance for the Apicomplexa orders. (c) Phylogenetic tree depicting the relationship between Apicomplexa ASVs. The bar plots at the tips of the branches represent the mean relative abundance of the ASV across the samples. Select groups with high mean relative abundances were annotated to the lowest known classification level.

The gregarines were the dominant group of Apicomplexa in the tropical soils surveyed, comprising 98% of all Apicomplexa reads (Table S2). Within the gregarines, taxa were composed mostly of the Eugregarinorida and Neogregarinorida (mean relative abundance = 68.6% and 21.0%, respectively; Figure 2b). Of the 189 Apicomplexa ASVs, 69 were Eugregarinorida and 75 were Neogregarinorida. The Gregarinomorphea (GRE7) comprised 31 ASVs. We also detected two orders within the class Coccidiomorphea, Adeleida (4 ASVs) and Eimeriida (3 ASVs), and several taxa unassigned past class (7 ASVs) (Figure 2c). Our assessment of the most abundant groups of soil Apicomplexa aligns with previous studies, and similar taxa were also found to be abundant in other cultivation‐independent surveys of soil Apicomplexa (Mahé et al. 2017; Li et al. 2023).

We next assessed how soil Apicomplexa from publicly available soil datasets (obtained using the metaPR2 database, Vaulot et al. 2022) phylogenetically clustered with ASVs from this study. First, we found that, not surprisingly, many of the ASVs from our study cluster with ASVs from another tropical soil study where samples were collected from similar sites in Panama (Mahé et al. 2017; Figure S1). Specifically, we identified six clades that appear to be restricted to tropical soils. There are two groups (Figure S1 and S1.2) within Eugregarinorida that represent tropical soil *Gregarina chortiocetes* and tropical *Gregarina tropica* clades, respectively. In Neogregarinorida there is a clade that consists of tropical soil *Monocystis* and *Syncystic* spp. (Figure S1.3), and Gregarinomorphea (GRE7) consists of two tropical soil clades (Figure S1.4 and S1.5). There was also a clade of tropical soil Adeleida apicomplexans (Figure S1.6). In addition to tropics‐specific clades, we also identified clades that were only detected in temperate soils (Seppey et al. 2020). For example, there is a temperate soil clade found within Eugregarinorida that consists of *Leidyana* sp. (Figure S1.7) and a clade within Neogregarinorida that consists of *Monocystis* and *Syncystic* spp. (Figure S1.8). Gregarines have been noted as the dominant group in both neotropical rainforests (representing ~80% of the Apicomplexa abundance; Mahé et al. 2017) and in subtropical rainforests (representing about ~90% of the Apicomplexa abundance; Li et al. 2023). Our findings underscore how variable Apicomplexa abundances are within tropical ecosystems and suggest that specific clades may be restricted to temperate or tropical habitats, although additional sequencing efforts will be valuable in confirming the preliminary trends we observe here with a limited number of datasets.

### 3.2 What Environmental Factors Predict the Diversity and Structure of Apicomplexa?

**FIGURE 3:**
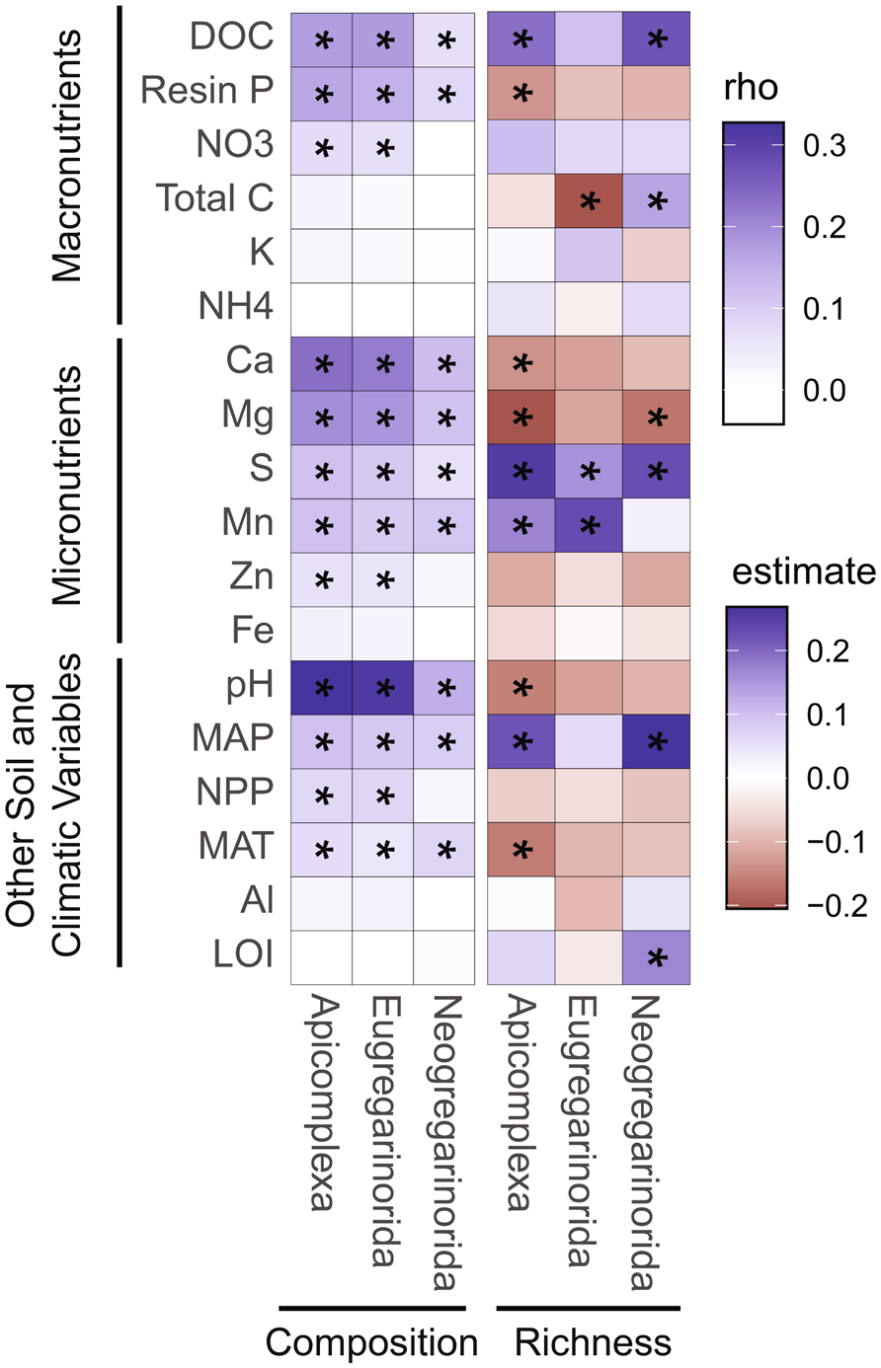
A heatmap representing the strength of Spearman correlations between the composition (Bray‐Curtis) and ASV richness of overall Apicomplexa, Eugregarinorida, and Neogregarinorida and a suite of abiotic variables. Asterisks (*) indicated significant correlation (*p* < 0.05).

Despite the considerable heterogeneity in the composition of Apicomplexa lineages, we found that the distribution of Apicomplexa communities was moderately related to specific environmental parameters. The overall composition of Apicomplexa (represented by Bray‐Curtis community dissimilarities) was most correlated to pH and Ca (rho = 0.33 and 0.34, respectively) and, to a lesser extent, Mg, DOC, available P, MAP, S, and Mn (*p* < 0.05 for all; Figure 3; Table S3). We then determined which subset of environmental parameters best explained the composition of Apicomplexa. The best model included soil pH, available P, and Mg (Table S4; *p* < 0.05; rho = 0.34). We also considered the two dominant lineages of Apicomplexa, Eugregarinorida and Neogregarinorida, separately, and found both subgroups were shaped by a similar set of abiotic parameters (Table S3), although the best model for Neogregarinorida included MAP and not available P (Table S4).

Our finding that pH is the most important environmental parameter in structuring Apicomplexa distributions is not surprising given the extensive research noting the importance of pH in structuring belowground bacterial and archaeal communities (Fierer et al. 2009), including in the tropics (Oliverio, Bissett, et al. 2020; de Carvalho et al. 2016; Tripathi et al. 2013). However, it does suggest that Apicomplexa are shaped by different factors than soil protists broadly, which are most strongly structured by MAP on a global scale (Oliverio, Geisen, et al. 2020; Bates et al. 2013) and may be less sensitive to precipitation. This is consistent with previous literature describing the environmental factors structuring soil parasites in temperate ecosystems. A survey of protists across UK soils also found that differences across pH gradients were largely driven by parasites and saprotrophs (Dupont et al. 2016). Similarly, a comparison of Apicomplexa distribution across land‐use habitats, Li et al. (2023) also uncovered a significant effect of pH on the composition of Apicomplexa communities.

Overall Apicomplexan richness was also moderately related to specific environmental parameters (Figure 3; Table S5), including S (rho = 0.25), MAP (rho = 0.22), and Mg (rho = −0.20) (see Figure 3; Table S5 for full outputs). Within the two main orders, both Eugregarinorida and Neogregarinorida richness were correlated to similar abiotic factors although Eugregarinorida richness was also strongly driven by Mn (rho = 0.23) and total C (rho = −0.20) and Neogregarinorida richness was strongly correlated to MAP (rho = 0.27) and DOC (rho = 0.22). Our findings align with previous assessments of Apicomplexa richness in other ecosystems which have also noted a positive correlation between Apicomplexa richness and MAP and DOC and a negative relationship with pH (Li et al. 2023). Understanding how Apicomplexa are distributed along environmental gradients is a key first step in understanding their potential contributions to soil and ecosystem processes.

### 3.3 To What Extent Do Apicomplexa Distributions Correspond to the Distribution of Soil Metazoa?

We first assessed to what extent the Apicomplexa richness and composition corresponded to Metazoa richness across the soils surveyed. Overall Apicomplexa richness did not correspond to overall metazoan richness (*p* = 0.63; rho = −0.03; Table S5), although Neogregarinorida‐specific richness was significantly correlated to both Annelida and Nematoda richness (*p* < 0.0.05; Figure S2; Tables S5 and S6). Other studies have detected similarity in Apicomplexa and Metazoa richness (Li et al. 2023; Singer et al. 2020). We also found a significant correlation between Apicomplexa and Metazoa community composition (rho = 0.17, *p* < 0.001). The correspondence between communities may be driven by shared environmental preferences or, in some cases, may indicate potential interactions between some Apicomplexa and Metazoa lineages in soil through host–parasite and host–symbiont relationships.

We then evaluated if the environmental parameters that shape Apicomplexa were similar for Metazoa soil communities. The strongest driver for the Metazoa composition (and the three major lineages surveyed: Annelida, Arthropoda, and Nematoda) was pH (rho = 0.29 for overall Metazoa; Figures S3 and S4; Table S6). As with Apicomplexa, soil pH, available P, and Mg were retained in the best model, along with S (Table S7). This is consistent with previous literature demonstrating that pH can shape tropical soil metazoan communities (Baker and Whitby 2003; Lakshmi and Joseph 2017; Karuri 2021). The model for Annelida also retained MAP, which is likely a reflection of the importance of water availability to their ecological distributions (Wever et al. 2001). Similarly to Apicomplexa richness, Metazoa richness was also significantly correlated with pH, Mg, and S (rho = 0.25, 0.24, and 0.15, respectively; Table S8). More broadly, these findings indicate that environmental parameters that shape Metazoa are similar to those that shape Apicomplexa.

To further assess the relationships between particular Apicomplexa and Metazoa taxa, we built ASV‐level co‐occurrence networks. We detected 3046 edges between all protists and Metazoa (*p* < 0.001) and specifically between Apicomplexa and Metazoa, we detected 72 edges (*p* < 0.001; Table S9). Our results are consistent with previous studies that have detected the co‐occurrence of gregarine taxa with arthropods, annelids, and nematodes (Sapkota et al. 2020; Wang et al. 2020; Li et al. 2023; Desportes and Schrével 2013). We also found connections between other soil metazoan groups, including Gastrotricha and Platyhelminthes, with Apicomplexa. This could indicate potential host–symbiont relationships with a broader set of soil metazoa than previously documented, or shared habitat preferences as discussed above. Apicomplexa are known to infect marine Platyhelminthes (Van Steenkiste et al. 2023) and it is likely they also associate with soil Platyhelminthes. Traditional taxonomy and biodiversity assays have often overlooked Gastrotricha due to a lack of knowledge and taxonomic expertise, and resource‐intensive methods for morphological‐based identification (Balsamo et al. 2020).

**FIGURE 4:**
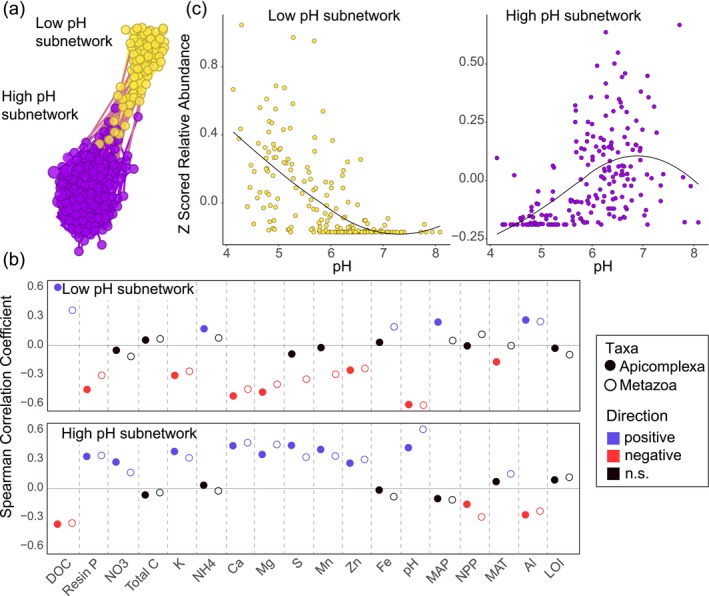
Summary of (a) the co‐occurrence subnetworks formed between protist and metazoan taxa showing (b) the Spearman correlations of the *z*‐scored relative abundances of Apicomplexa and Metazoa taxa. Positive significant correlations are indicated by blue circles, negative significant correlations are indicated by red circles, and nonsignificant (*p* > 0.05) are indicated by black circles. A scatter plot with a regression line illustrating the (c) direction of the relationship between the *z*‐scored relative abundances of all organisms in a subnetwork and pH.

Using a modularity analysis, we identified two distinct subnetworks (i.e., distinct modules in which significant correlations between ASVs were more abundant within the subnetworks than across the two subnetworks; Figure 4a). The relative abundances of the taxa within the first subnetwork (i.e., the low pH module) were negatively related to pH, Ca, and Mg (Figure 4b,c; Table S9). Conversely, taxa in the second subnetwork (i.e., the high pH module) were significantly positively correlated with these variables (Figure 4b,c; Table S9). Beyond co‐correspondence with environmental variables delineating the two Apicomplexa/Metazoa subnetworks, there were differences in the connections between taxa (Table S10). The low pH subnetworks contained more Apicomplexa‐Nematoda (*N* = 14) and Apicomplexa‐Arthropoda connections (*N* = 6), whereas Apicomplexa‐Annelida connections (*N* = 5) were more prevalent in the high pH subnetwork. Li et al. (2023) likewise found that increasing edaphic preferences (specifically soil moisture) increased the probability of apicomplexan association with metazoans (Annelida, Arthropoda, and Nematoda). Overall, these results advance our understanding of potential environmental niche preferences for host–parasite biogeography.

We also assessed to what extent Apicomplexa may co‐occur with other protist lineages. We detected 285 edges (*p* < 0.001; Table S11) including connections between the Apicomplexan groups and Cercozoa, Ciliophora, Chlorophyta, Chrompodellids, Conosa, Foraminifera, Lobosa, Ochrophyta, Pseudofungi, Sagenista, and Discoba. Over half of the connections found were specific to Cercozoans (144/285 edges), Ciliophora (38 connections), and Conosa (51 connections). Although there is limited support to describe and interpret these potential relationships, it is notable that these protist groups co‐occur. Future research would benefit from untangling the potential interactions between Apicomplexa and other protists.

### 3.4 Caveats and Future Directions

Our findings shed light on the diversity and biogeography of Apicomplexa lineages in tropical soils; however, we note that there are important limitations in analyses of environmental DNA. First, the taxonomic resolution of both Apicomplexa and Metazoa remains limited due to a lack of representation in sequence databases (Gao et al. 2024; del Campo et al. 2019; Bik 2019), so there is bias toward some groups such as arthropod families that have more representation in databases. The issue of taxonomic resolution in this study may be further confounded by our limitation of only utilizing the forward reads. Second, there are substantial differences in body sizes across Metazoan lineages, which can bias the detection and estimates of relative abundance. Nevertheless, metabarcoding approaches still yield strong correspondence with morphological approaches of assessing soil diversity of Arthropods (Oliverio et al. 2018). Third, our DNA‐based study did not allow us to determine which Apicomplexa were active (likely recovered from parasitized soil invertebrates) versus encysted in soils. Future efforts may benefit characterization of Apicomplexa from individual arthropods, nematodes, annelids, or other soil fauna to help resolve host–specific relationships. These limitations of creating direct links between Apicomplexan parasites and their hosts are echoed by other studies that utilize 18S sequencing (Holt et al. 2022). Co‐occurrence analyses cannot confirm biotic interactions but may indicate potential host–parasite interactions (Blanchet et al. 2020). Likewise, using RNA‐based methods to profile Apicomplexa in soil studies may help distinguish the active community versus resistant cells (e.g., encysted) in the soil environment (Rueckert et al. 2011; Dupont et al. 2016).

Our findings highlight the importance of soil pH in structuring both Apicomplexa and the major Metazoan lineages they parasitize in belowground tropical systems. Despite the highly heterogeneous distribution of Apicomplexa lineages across the soils surveyed, overall composition was moderately correlated with both environmental variables (namely, pH) and Metazoan lineages. This suggests that Apicomplexa are structured by different factors than overall soil protist communities and may be less sensitive to soil moisture than other protists, perhaps due to their ecological role as host‐associated parasites. Our detection of specific Apicomplexa and Metazoa that co‐occur may indicate putative host–symbiont relationships or shared patterns of distribution due to similar abiotic preferences in habitat. Broadly, our efforts begin to define the biogeographic drivers of parasites in tropical soil ecosystems and to integrate belowground soil parasites and their hosts into our broader understanding of soil microbiomes.

## Author Contributions

**Rachel M. Shepherd:** conceptualization (equal), data curation (lead), formal analysis (lead), visualization (lead), writing – original draft (equal), writing – review and editing (equal). **Angela M. Oliverio:** conceptualization (equal), data curation (supporting), formal analysis (supporting), funding acquisition (lead), writing – original draft (equal), writing – review and editing (equal).

## Conflicts of Interest

The authors declare no conflicts of interest.

## Supporting information


**Figure S1.** Phylogenetic tree depicting the relationship between Apicomplexa ASVs from the present study, Mahe et al., 2017 (tropical soils), Seppey et al., 2020 (temperate/Swiss soils), and Bates et al., 2013 (global soils).
**Figure S2.** A heatmap representing the strength of Spearman correlations between the composition (Bray‐Curtis) and ASV richness of overall Apicomplexa, Eugregarinorida, and Neogregarinorida and the composition (Bray‐Curtis) and ASV richness of overall Metazoa, Annelida, Arthropoda, and Nematoda.
**Figure S3.** A heatmap representing the strength of Spearman correlations between the composition (Bray‐Curtis) and ASV richness of overall Metazoa, Annelida, Arthropoda, and Nematoda and a suite of abiotic variables.
**Figure S4.** NMDS of (a) Apicomplexa composition (Bray‐Curtis) and (b) NMDS of Metazoa community composition (Bray‐Curtis).

**Table S1.** Metadata of all samples (*N* = 205) used in this paper.
**Table S2.** Mean relative abundance of Apicomplexa taxa across 171 samples.
**Table S3.** The output of Spearman correlations between the community composition (Bray‐Curtis) of overall Apicomplexa, Eugregarinorida, and Neogregarinorida and a suite of abiotic and biotic variables.
**Table S4.** The output of the best subset of variables explaining the community composition (Bray‐Curtis) of overall Apicomplexa, Eugregarinorida, and Neogregarinorida.
**Table S5.** The output of Spearman correlations between the richness of overall Apicomplexa, Eugregarinorida, and Neogregarinorida and a suite of abiotic and biotic variables.
**Table S6.** The output of Spearman correlations between the community composition (Bray‐Curtis) of overall Metazoa, Annelida, Arthropoda, and Nematoda and a suite of abiotic and biotic variables.
**Table S7.** The output of the best subset of variables explaining the community composition (Bray‐Curtis) of overall Metazoa, Annelida, Arthropoda, and Nematoda.
**Table S8.** The output of Spearman correlations between the richness of overall Metazoa, Annelida, Arthropoda, and Nematoda and a suite of abiotic and biotic variables.
**Table S9.** The output of Spearman correlations between the *z*‐scored relative abundances of Apicomplexa and Metazoa and a suite of abiotic variables for the two distinct subnetworks.
**Table S10.** The sum of edges (or connections) between Apicomplexa and Metazoa taxa in the two subnetworks.
**Table S11.** The sum of edges (or connections) between Apicomplexa and other protist taxa in the two subnetworks.

## Data Availability

Sequence data are available under PRJNA1167728. Data corresponding to this study are on Figshare under https://doi.org/10.6084/m9.figshare.27251949.
