# Supplementary material for: The Biogeography of Apicomplexan Parasites in Tropical Soils
Source: Ecol Evol. 2025 Jun 2;15(6):e71478. doi: 10.1002/ece3.71478 (PMC12130279; doi:10.1002/ece3.71478)

TITLE: The biogeography of Apicomplexan parasites in tropical soils

RUNNING TITLE: Apicomplexa in tropical soils

AUTHORS: Rachel M. Shepherd[1] and *Angela M. Oliverio[1]

1. Syracuse University, Department of Biology, Syracuse, NY
*Corresponding author: amoliver@syr.edu

Syracuse University Department of Biology
Life Sciences Complex, 107 College Pl, Syracuse, NY 13210

SI Figure 1. Phylogenetic tree depicting the relationship between Apicomplexa ASVs from the present study, Mahe et al., 2017 (tropical soils), Seppey et al., 2020 (temperate/Swiss soils), and Bates et al., 2013 (global soils). Numbers indicate highlighted clades: 1) Tropical *Gregarina chortiocetes* clade, 2) Tropical *Gregarina tropica* clade, 3) Tropical *Monocystis* and *Syncystis* clade, 4) Tropical Gregarinomorphea (GRE7) clade, 5) Tropical Gregarinomorphea (GRE7) clade, 6) Tropical Adeleida clade, 7) Temperate *Leidyana sp*. clade, and 7) Temperate *Monocystis* and *Syncystis* clade.

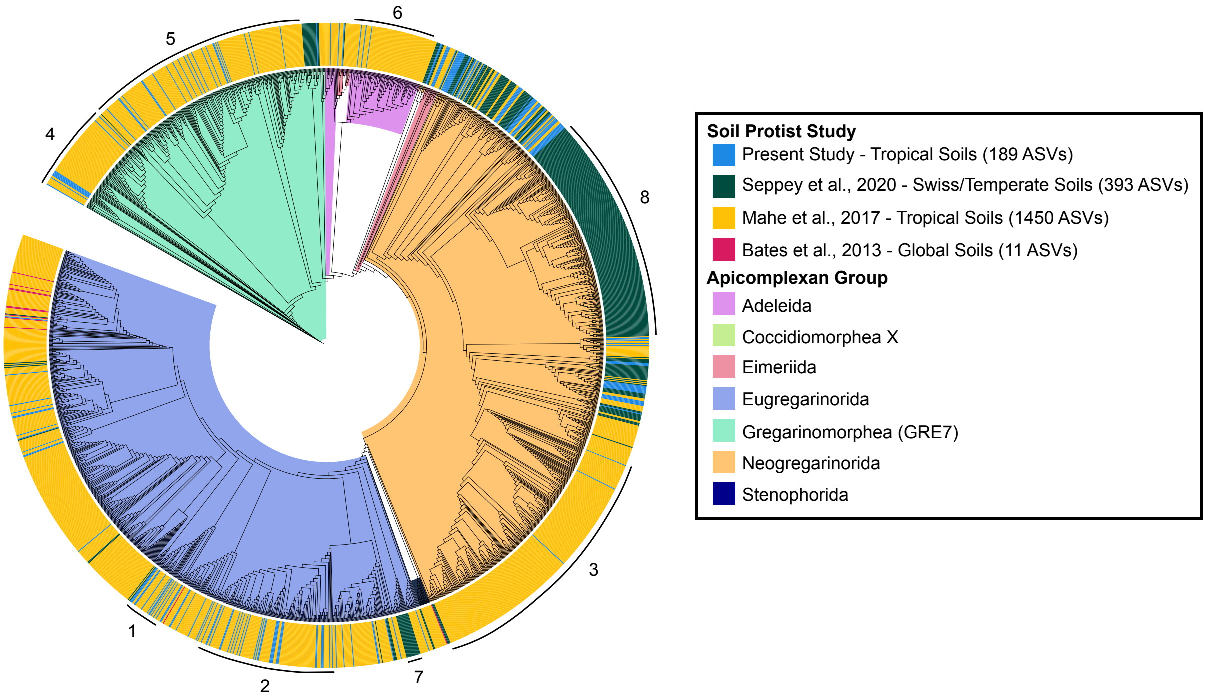

SI Figure 2. A heatmap representing the strength of Spearman correlations between the composition (Bray-Curtis) and ASV richness of overall Apicomplexa, Eugregarinorida, and Neogregarinorida and the composition (Bray-Curtis) and ASV richness of overall Metazoa, Annelida, Arthropoda, and Nematoda. Asterisks (*) indicated significant correlation (*P* < 0.05).

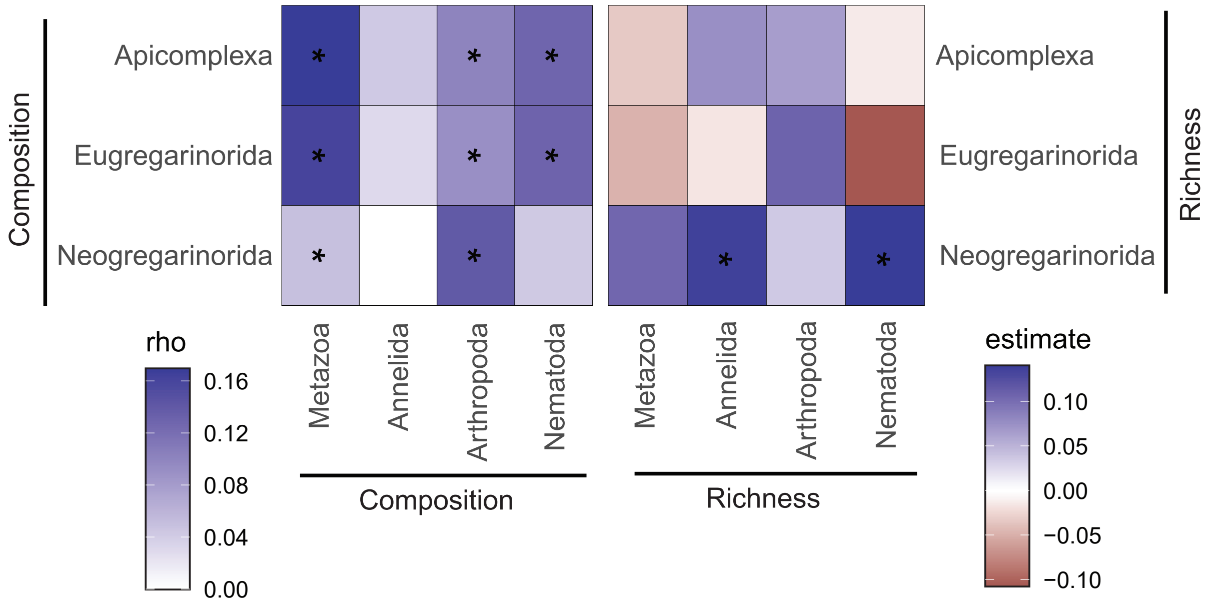

SI Figure 3. A heatmap representing the strength of Spearman correlations between the composition (Bray-Curtis) and ASV richness of overall Metazoa, Annelida, Arthropoda, and Nematoda and a suite of abiotic variables. Asterisks (*) indicated significant correlation ($P < 0.05$).

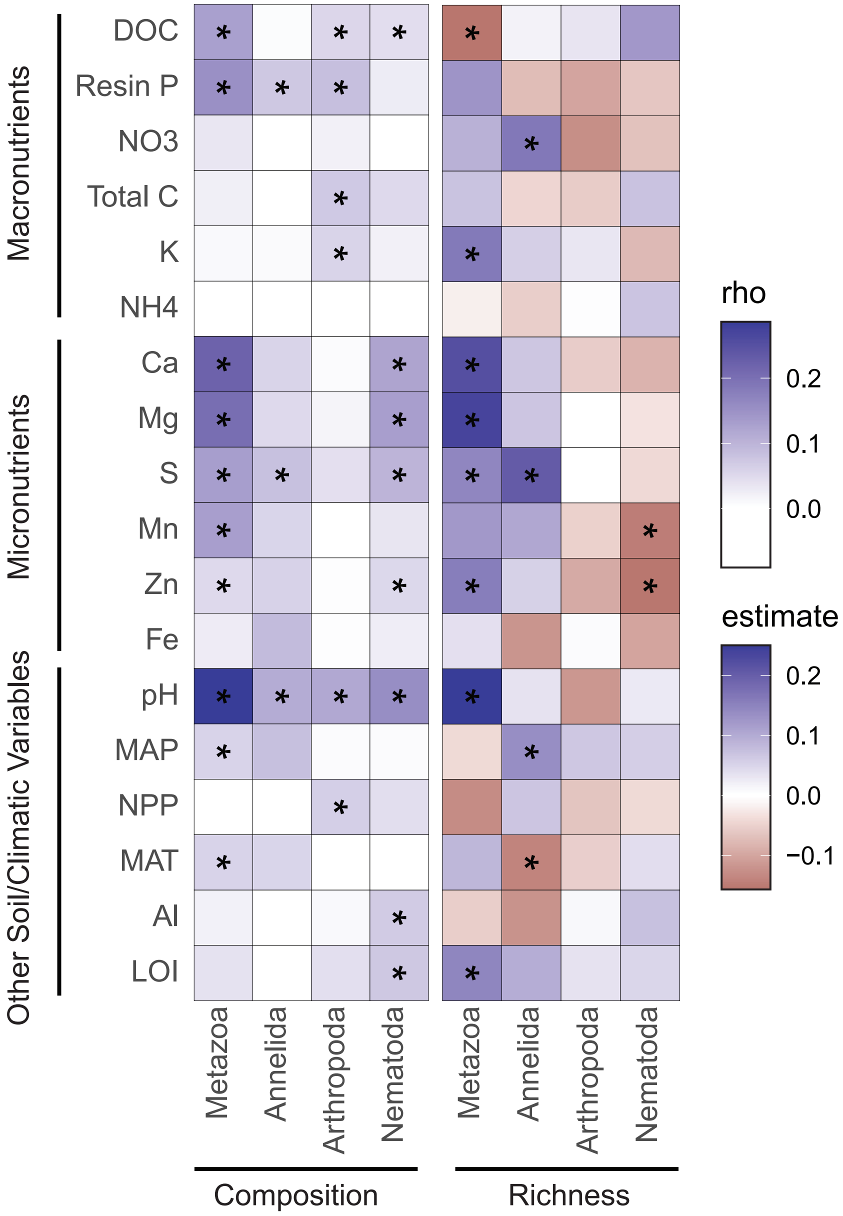

SI Figure 4. NMDS of **(a)** Apicomplexa composition (Bray-Curtis) and **(b)** NMDS of Metazoa community composition (Bray-Curtis).

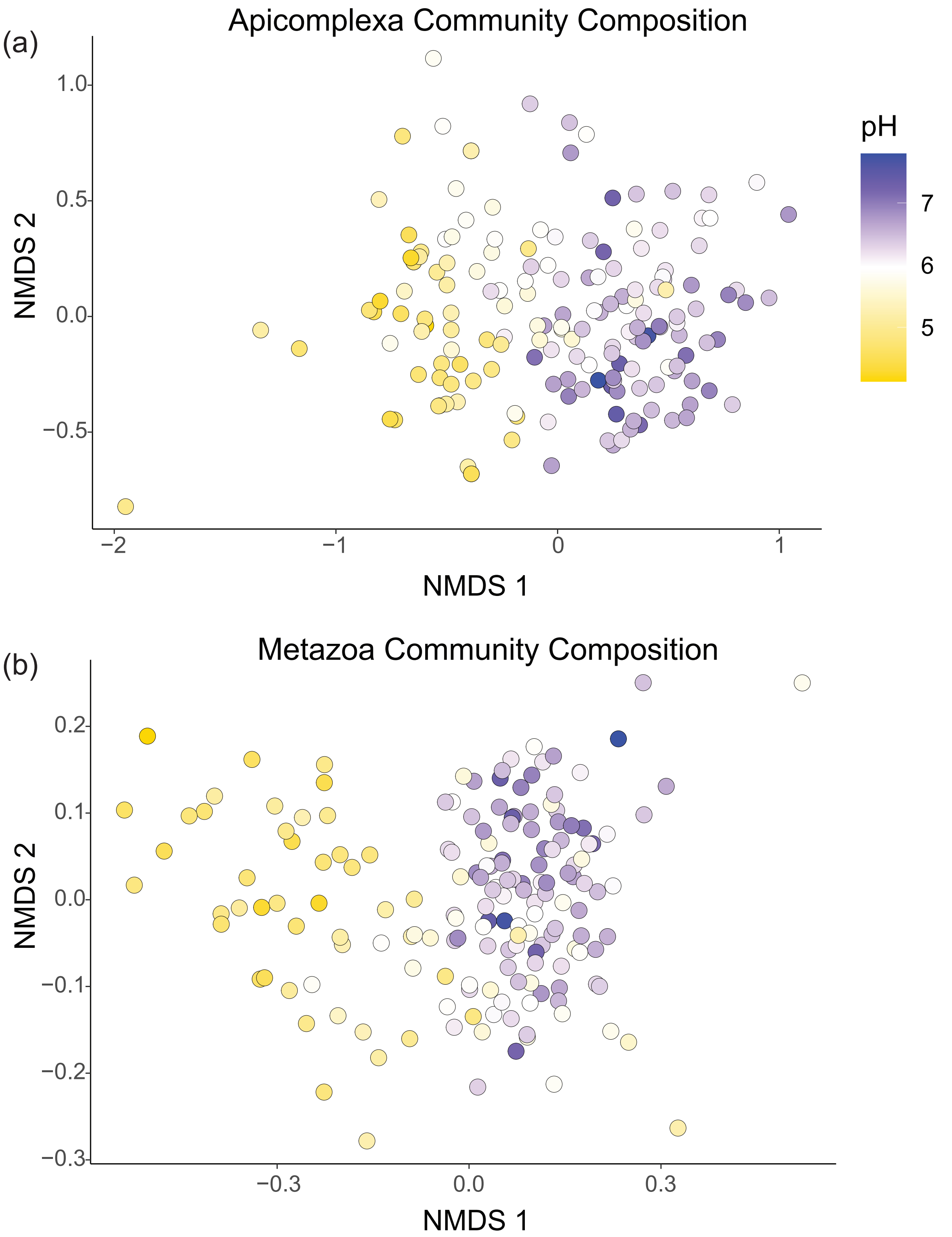

Supplement: Supplementary file 1 — Figure S1. Phylogenetic tree depicting the relationship between Apicomplexa ASVs from the present study, Mahe et al., 2017 (tropical soils), Seppey et al., 2020 (temperate/Swiss soils), and Bates et al., 2013 (global soils). Figure S2. A heatmap representing the strength of Spearman correlations between the composition (Bray‐Curtis) and ASV richness of overall Apicomplexa, Eugregarinorida, and Neogregarinorida and the composition (Bray‐Curtis) and ASV richness of overall Metazoa, Annelida, Arthropoda, and Nematoda. Figure S3. A heatmap representing the strength of Spearman correlations between the composition (Bray‐Curtis) and ASV richness of overall Metazoa, Annelida, Arthropoda, and Nematoda and a suite of abiotic variables. Figure S4. NMDS of (a) Apicomplexa composition (Bray‐Curtis) and (b) NMDS of Metazoa community composition (Bray‐Curtis). [file ECE3-15-e71478-s002.pdf]
